# Supplementary material for: Proteomic Profiling Differentiates Lymphoma Patients with and without Concurrent Myeloproliferative Neoplasia
Source: Cancers (Basel). 2021 Nov 3;13(21):5526. doi: 10.3390/cancers13215526 (PMC8583469; doi:10.3390/cancers13215526)
Supplement: Supplementary file 1 [file cancers-13-05526-s001.zip › cancers-1429931-supplementary.pdf]

cancers MDPI

Supplementary Material: Proteomic Profiling Differentiates Lymphoma Patients with and without Concurrent Myeloproliferative Neoplasia

Johanne Marie Holst, Marie Beck Enemark, Martin Bjerregaard Pedersen, Kristina Lystlund Lauridsen, Trine Engelbrecht Hybel, Michael Roost Clausen, Henrik Frederiksen, Michael Boe Møller, Peter Nørgaard, Trine Lindhardt Plesner, Stephen Jacques Hamilton-Dutoit, Francesco d'Amore, Bent Honoré and Maja Ludvigsen

Supplementary methods

FFPE tissue preparation

Proteins from FFPE tissue samples of lymphoid origin were extracted from seven 10µm thick sections as previously described [1]. Paraffin was removed with xylene and the tissue was rehydrated with graded concentrations of ethanol and water and vacuum dried. The dried tissue was mixed with lysis buffer (5% sodium deoxycholate (SDC), 20 mM triethylammonium bicarbonate (TEAB)), and incubated in a heating block with agitation at 99 °C for one hour. The suspension was homogenized and sonicated on ice. Following centrifugation, the protein concentration in the supernatant was measured by infrared spectrometry [1]. The proteins were reduced by tris(2-carboxyethyl)phosphine (200 mM) followed by incubation at 55°C for one hour and alkylated with iodoacetamide (375mM) followed by incubation in the dark at room temperature for 30 minutes. Proteins were digested with Pierce™ Trypsin Protease, MS Grade (ThermoFisher Scientific, Massachusetts, USA) overnight in a wet chamber at 37 °C. SDC was removed by phase transfer with ethyl acetate and acidification with trifluoroacetic acid. The peptide amount was measured by fluorescence [1] and an amount of 250ng of each sample was analyzed in duplicate by label-free quantification nano liquid chromatography - tandem mass spectrometry (LFQ nLC-MS/MS).

LFQ nLC-MS/MS

The peptide mixtures were separated by nano Liquid-Chromatography (Ultimate 3000, Dionex) coupled to a mass spectrometer (Orbitrap Fusion, ThermoFisher Scientific) through an EASY-Spray nano-electrospray ion source (ThermoFisher Scientific). A - Precolumn (300µm x 5mm, C18 PepMap100, 5µm, 100Å, ThermoFisher Scientific) and analytical column (EASY-Spray Column, 500mm x 75µm, PepMap RSCL, C18, 2mm, 100Å, ThermoFisher Scientific) were used to trap and separate peptides, respectively. The peptides were eluted with a flow of 300nL/min. using a 213 min. gradient by mixing buffer A (99.9% water, 0.1% formic acid) with buffer B (99.9% acetonitrile, 0.1% formic acid). The following amount of buffer B was used: 2% (0 min.), 12% (3 min.), 25% (149 min.), 40% (156 min.), 80% (159 min.), 80% (175 min.), 2% (176 min.) and the LC was run until 213min. The universal method setting was used for mass spectrometry detection for 184 min. starting at 0 min. with settings as previously described [2]. Internal mass calibration was used by activating the EASY-IC using fluoranthene.

Database searches

The raw data files were used to search the human database from UniProt (downloaded on the 10.04.2018) and using MaxQuant (version 1.5.5.1) for LFQ analysis [3]. The settings were as previously described [4]. The generated results file was then entered into Perseus (v 1.6.2.3) for further analysis [5] where data was filtered and Log_2 transformed. The median technical coefficient of variation of all the protein levels in each sample was between 5.56% and 12.63% with a mean of 7.41%. The means of the technical replicates were used to calculate the protein amount in samples.

Immunohistochemical staining of selected proteins

Whole biopsy 4µm FFPE tissue sections were loaded onto the Ventana BenchMark ULTRA automated slide stainer (Ventana Medical Systems, Roche, Oro Valley, AZ, USA) and were immunohistochemically stained by standard methods using the OptiView DAB IHC Detection Kit (Ventana Medical Systems, Roche). Heat induced epitope retrieval was applied by heating to 100°C for 32 minutes. Polyclonal rabbit anti-human antibody against isocitrate dehydrogenase 2 (*IDH2*) (product no. HPA007831, Sigma-Aldrich, Missouri, USA, dilution 1:100), DnaJ homolog subfamily A member 2 (*DNAJA2*) (product no. HPA060538, Sigma-Aldrich, dilution 1:100), citrate synthase (product no. HPA038461, Sigma-Aldrich, dilution 1:100), lactotransferrin (product no. HPA059976, Sigma-Aldrich, dilution 1:3000) and myeloblastin (product no. HPA005938, Sigma-Aldrich, dilution 1:300) were diluted in Tris buffered antibody diluent (pH 7.2, 15mmol/L NaN_3 and stabilizing protein, Dako, CA, USA), followed by 32 minutes of incubation at 36°C. Sections of appendix, tonsil, liver, and pancreas were included on each slide as external controls.

Digital image analysis

Stained slides were scanned at a magnification of 20x using the Hamamatsu Nanozoomer 2.0HT scanner (Hamamatsu Photonics, Shizuoka, Japan), creating digital images of the stained whole tissue sections. Before applying digital staining classification, the region of interest (ROI) was defined by manual evaluation of all digitalized images, assuring that large areas of non-lymphoid tissue and preanalytical artefacts were excluded. Staining quantification was performed within the ROI using Visiopharm Integrator System 2019.12 (Visiopharm A/S, Hoersholm, Denmark). An application package protocol (APP) was designed for each marker by manual designation of image classes to appropriate tissue areas (*i.e.* weak-, intermediate-, and strong intensity staining as well as tissue background). The APP was trained on tissue sections representing different expression levels of the marker. The quantification was performed as area fractions (AFs), defined as the stained area normalized to the total area within the ROI. Expression levels of *IDH2* protein were based on AFs of the sum of strong and intermediate intensity staining. Expression levels of *DNAJA2* protein, citrate synthase, lactotransferrin and myeloblastin were based on AFs of all positive staining.

Supplementary figures

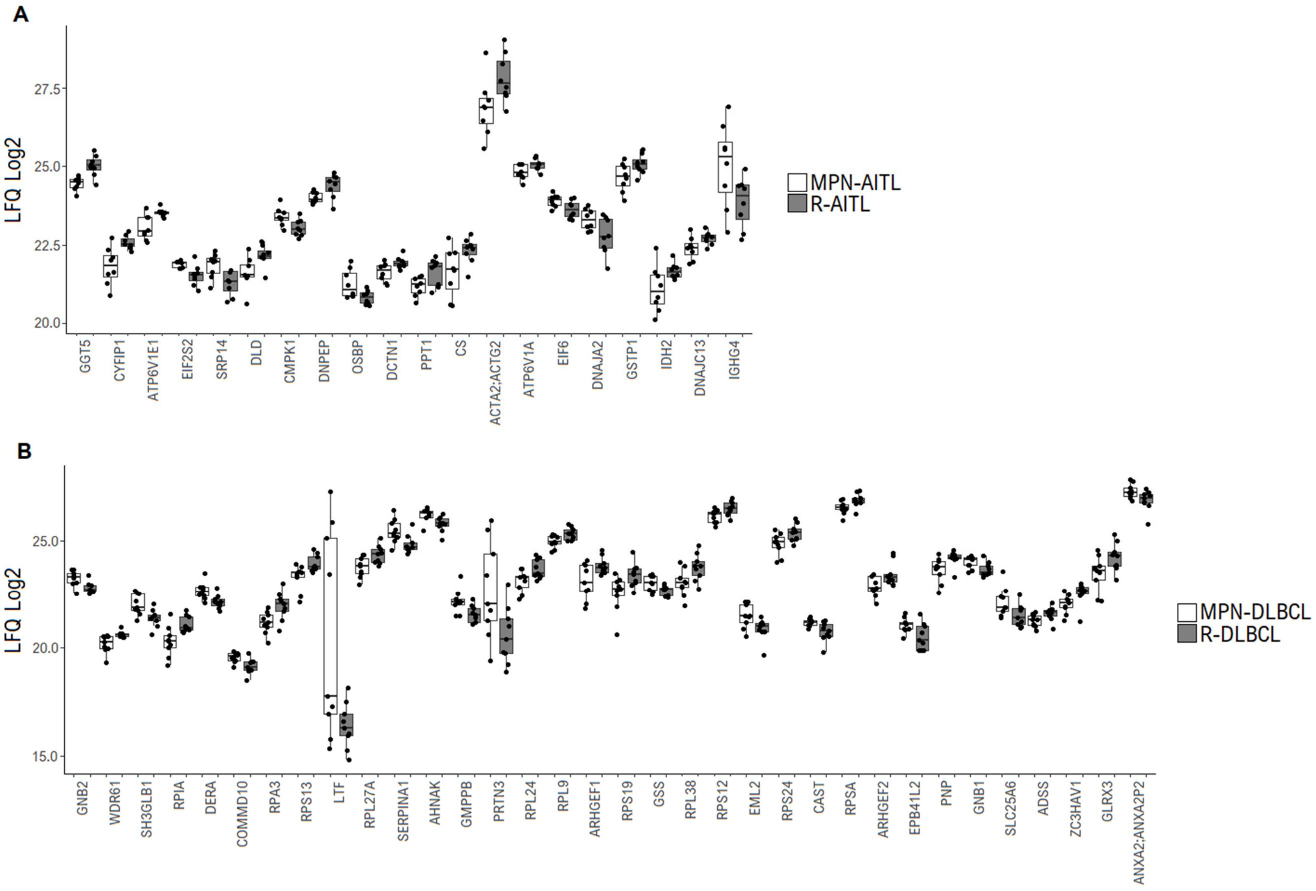

Figure S1. Expression of significantly differentially expressed proteins based on the proteomic analyses. (**A**) Expression levels of the 20 identified significantly differentially expressed proteins in the MPN-AITL and R-AITL tumors, respectively. (**B**) Expression levels of the 34 identified significantly differentially expressed proteins in the MPN-DLBCL and R-DLBCL tumors, respectively. Abbreviations: AITL, angioimmunoblastic T-cell lymphoma; DLBCL, diffuse large B-cell lymphoma; MPN, myeloproliferative neoplasia; R-, reference sample.

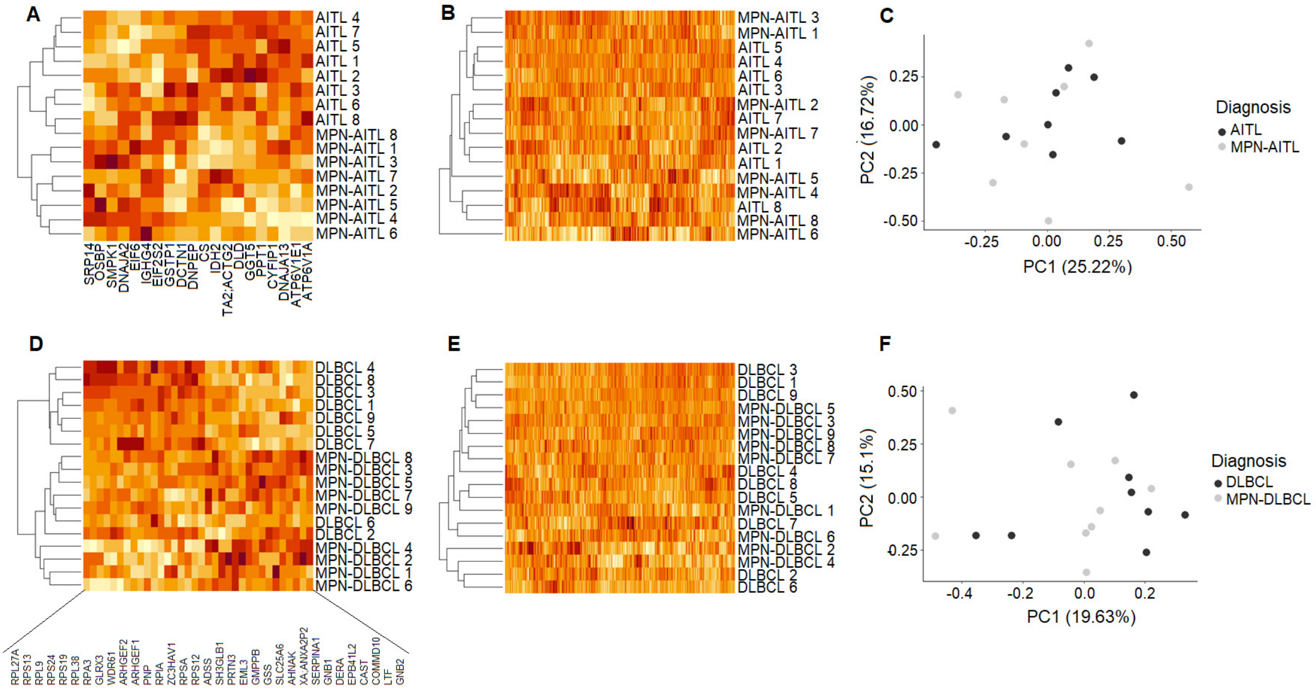

Figure S2. Clustering of patients based on significantly differentially expressed proteins and all identified differentially expressed proteins, respectively. (A) Heatmap and hierarchal clustering with input of the 20 significantly differentially expressed proteins between the MPN-AITL and R-AITL tumors. (B) Heatmap and hierarchal clustering with input of all 1074 identified differentially expressed proteins between the MPN-AITL and R-AITL tumors. (C) PCA with input of all 1074 differentially expressed proteins between the AITL and R-AITL tumors. (D) Heatmap and hierarchal clustering with input of the 34 significantly differentially expressed proteins between the MPN-DLBCL and R-DLBCL tumors. (E) Heatmap and hierarchal clustering with input of all 1141 identified differentially expressed proteins between the MPN-DLBCL and R-DLBCL tumors. (F) PCA with input of all 1141 differentially expressed proteins between the DLBCL and R-DLBCL tumors. Abbreviations: AITL, angioimmunoblastic T-cell lymphoma; DLBCL, diffuse large B-cell lymphoma; MPN, myeloproliferative neoplasia; PCA, principal component analysis; R-, reference sample.

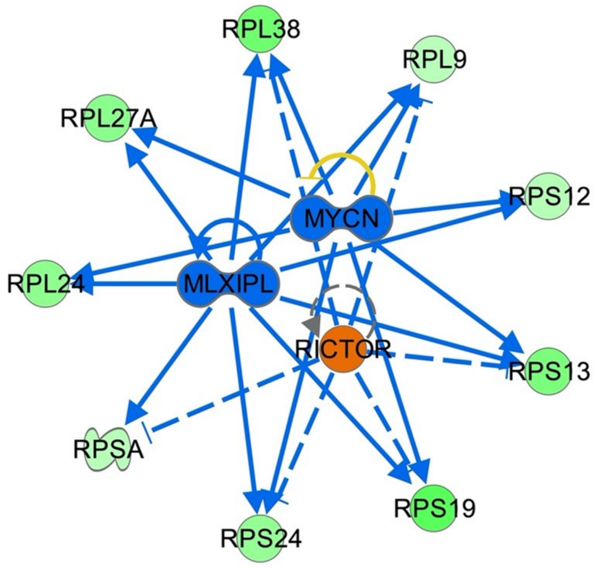

Figure S3. Ingenuity pathway analysis of differentially expressed proteins between MPN-DLBCL and R-DLBCL. The observed downregulation of nine ribosomal proteins (shown in green) in the periphery could be explained by activity changes in proteins shown in the inner circle. Thus, decreased activity of the transcription factors *MLXIPL* or *MYCN or* increased activity of *RICTOR* could contribute to explain the significant downregulation of the "EIF2 signaling" pathway. Solid lines: direct relationship. Stippled lines: indirect relationship. Blue lines: resulting in inhibition. Grey line: the effect cannot be predicted. Green color: increasing green color intensity indicates more decreased protein expression. Abbreviations: DLBCL, diffuse large B-cell lymphoma; MPN, myeloproliferative neoplasia; R-, reference sample.

References

1. Honoré B. Proteomic Protocols for Differential Protein Expression Analyses. *Methods Mol Biol.* 2020;2110:47-58.
2. Ludvigsen M, Thorlacius-Ussing L, Vorum H, et al. Proteomic Characterization of Colorectal Cancer Cells versus Normal-Derived Colon Mucosa Cells: Approaching Identification of Novel Diagnostic Protein Biomarkers in Colorectal Cancer. *Int J Mol Sci.* 2020;21(10).
3. Tyanova S, Temu T, Cox J. The MaxQuant computational platform for mass spectrometry-based shotgun proteomics. *Nat Protoc.* 2016;11(12):2301-2319.
4. Christakopoulos C, Cehofski LJ, Christensen SR, Vorum H, Honoré B. Proteomics reveals a set of highly enriched proteins in epiretinal membrane compared with inner limiting membrane. *Exp Eye Res.* 2019;186:107722.
5. Tyanova S, Temu T, Sinitcyn P, et al. The Perseus computational platform for comprehensive analysis of (prote)omics data. *Nat Methods.* 2016;13(9):731-740.
